# Current rehabilitation for adults with peripheral nerve injuries in the UK: An online survey

**DOI:** 10.1177/17589983261420313

**Published:** 2026-02-04

**Authors:** Caroline Miller, Suzanne Beale, Beth Fordham, David J. Keene, Kenneth F. Valyear

**Affiliations:** 1 1724 University of Birmingham, Birmingham, UK; 2 University Hospitals Birmingham, NHS Foundation Trust, Birmingham, UK; 3 6396 University of Oxford, Oxford, UK; 4 Bangor University, Wales, UK

**Keywords:** peripheral nerve injury, rehabilitation, therapy, brachial plexus injury, psychology, clinical psychology

## Abstract

**Introduction:**

Peripheral nerve injuries can lead to paralysis, sensory loss, chronic pain, and profound psychological and vocational consequences. Recent UK guidelines recommend biopsychosocial rehabilitation, yet qualitative evidence suggest gaps in service provision. This study explored current rehabilitation and perceived barriers among therapists treating adults with peripheral nerve injuries.

**Methods:**

A cross-sectional online survey was distributed to UK physiotherapists and occupational therapists experienced in upper and lower limb peripheral nerve injury rehabilitation. Questions captured demographics, treatment strategies before and after reinnervation, access to psychological support, and perceived organisational barriers. Descriptive analysis was undertaken.

**Results:**

Fifty-three respondents (60% physiotherapists, 40% occupational therapists) completed the survey; 70% had more than 10 years’ experience. Ninety eight percent of respondents treated adults with upper limb nerve injuries. Motor and sensory interventions (active and passive range of motion, splinting, strength training, and functional activity) were reported as “always/frequently” used by >80% of respondents. Over a quarter of respondents (28%) occasionally/rarely used pain neuroscience education. Other specific psychological interventions including cognitive behavioural techniques and mindfulness were rarely used (<30% always/frequently). Over half respondents reported no outpatient access to clinical psychology. Organisational barriers included limited time, funding, skilled staff, and absence of local guidelines. However, 83% believed therapists could deliver more psychologically informed care.

**Conclusions:**

UK rehabilitation for adults with peripheral nerve injuries remains predominantly biomedical, with limited integration of psychologically informed care and limited access to Clinical Psychology. Addressing systemic barriers and evaluating digital or hybrid models may enable more biopsychosocial, patient-centred care.

## Introduction

Peripheral nerve injuries can lead to life-changing consequences including paralysis, sensory loss, chronic pain, and substantial effects on psychological well-being and employment.^1,2^ A nerve injury is frequently associated with traumatic limb injury, with the upper limb most commonly affected.^3^ Adults with nerve injuries are, on average, six times more likely to develop chronic pain than those with traumatic injuries without nerve involvement.^2^ Psychological consequences are also profound, with 91% of adults with nerve injury presenting with post-traumatic stress at 1 month.^4^ Depression, stress, reduced pain self-efficacy, and sleep disturbance are also strongly predictive of poor functional outcome in adults with nerve injury.^1,5^ Nerve injuries disproportionately affect adults in the peak of their working lives, with men twice as likely as women to be affected^6,7^ and those from lower socioeconomic backgrounds 5–10 times more likely to sustain an upper limb nerve injury.^8^ Finally, more than 40% of individuals with severe upper limb nerve injury remaining out of work after 1 year.^9^

To address these needs, recent NICE guidelines^10^ recommend a biopsychosocial approach to rehabilitation following traumatic nerve injury. First described by Engel in 1977, the biopsychosocial model proposes that health and recovery are shaped by the dynamic interaction between biological processes (e.g., nerve regeneration, pain mechanisms), psychological factors (e.g., distress, coping, pain self-efficacy) and social influences (e.g., work, family socioeconomic context).^11^ Applied to traumatic nerve injury it suggests that optimal recovery cannot be achieved through physical rehabilitation alone: psychological factors, social determinants must also be addressed to optimise outcomes.

However, there is evidence of a mismatch between these recommendations and patient experience. Some adults with severe nerve injuries in the UK have reported struggling to cope following the injury due to inconsistent information and limited access to appropriate rehabilitation.^12,13^ In a recent patient consensus meeting, people with severe upper limb nerve injuries identified “access to appropriate treatment” as one of their top priorities for improving outcome following the injury.^12^ Similarly, qualitative research from Sweden found that adults with traumatic brachial plexus injury (a severe upper limb nerve injury) described difficulties in accessing person-centred rehabilitation.^14^

Although qualitative research and consensus work with adults with nerve injury highlight challenges in access to appropriate rehabilitation and it’s potential variability, it remains unclear how this rehabilitation is delivered across the UK; and the extent to which psychological needs are addressed. Understanding current rehabilitation provision, alongside perceived system barriers and facilitators, is essential to inform service development and future best practice guidelines for this population.

The aim of this study was to:

(1) Describe current rehabilitation practice (physiotherapy, occupational therapy, and psychological support) for people with peripheral nerve injury, including brachial plexus injury, in the UK.
(2) Explore healthcare professionals’ perceptions of system barriers and facilitators to rehabilitation in this population.

## Methods

This study was approved by the Ethics Committee of the School of Psychology & Sport Science at University of Bangor (Reference: 0630).

### Questionnaire development and piloting

A cross-sectional online survey, hosted by Microsoft Forms (Microsoft Office 365), was developed using a Consensus-Based Checklist for Reporting of Survey Studies (CROSS).^15^ The web-based questionnaire was designed to capture current UK peripheral nerve injury rehabilitation provision from healthcare professionals and perceived barriers and facilitators. The patient population which therapists were asked to consider were traumatic peripheral nerve injuries (excluding digital nerve injuries), therefore focusing on more severe nerve injuries. Participants were asked to consider rehabilitation for patients treated non-surgically in addition to those following primary surgical interventions including nerve repair and nerve grafting. Upper and lower limb nerve injuries were included as NICE guidelines on rehabilitation after traumatic injury^10^ include both and a recent epidemiological study^6^ in England includes both. Also nerve injury units, predominantly hosted in/with hand therapy centres, treat both upper and lower nerve injuries (Leeds, Birmingham, Manchester, Glasgow).

The online survey was developed by researchers, physiotherapists and occupational therapists with experience in survey development as there were no existing validated tools designed to capture the data required. The questionnaire was developed using items from literature searching and from clinical experience to populate suggested rehabilitation strategies. Previously published musculoskeletal surveys^16,17^ influenced the structure, including the type of demographic data collected and use of Likert-type scales. The questionnaire was piloted with healthcare professionals (Physiotherapist x 2, Occupational Therapist x1 and Nurse x1) and edits were made in response to their feedback. A health psychologist reviewed the survey, and amendments were made in response to their feedback.

### Survey content

The survey consisted of thirty questions in five sections: eligibility, demographics, experience and preferred treatment options, barriers to rehabilitation and current access to psychological support. The design was similar to previous musculoskeletal healthcare professional surveys evaluating current practice^18,19^ and included questions with a combination of response options including dichotomous yes/no answers, multiple choice, 5-point Likert scales and some questions had free-text options. The sections investigating preferred treatment strategies used the timeframes before and after sensory and motor reinnervation to examine potential changes in practice between these two distinct phases in nerve regeneration. A five-point Likert-type scale was used (Always, Frequently, Occasionally, Rarely, Never). Respondents were asked to rate their agreement on a list of potential barriers to rehabilitation for adults with traumatic nerve injuries via a five-point Likert scale (Strongly agree, Agree, Disagree, Strongly disagree, Neutral). Current access to psychological support was evaluated using dichotomous yes/no questions and fixed waiting time options. The online survey was anonymous. We did not collect any identifiable data such as names or age or name of place of work. A full copy of the survey is available in Supplemental Material (S1).

### Recruitment

The target population was Health and Care Professions Council registered occupational therapists and physiotherapists with self-reported experience in treating adults with peripheral nerve injury in the UK. Exclusion criteria included non-registered therapists or therapists without experience in rehabilitation of adults with a peripheral nerve injury.

The British Association of Hand Therapy and The Association of Trauma and Orthopaedic Physiotherapists in the UK disseminated a link to the survey with an embedded Participant Information Sheet to their members. The study was advertised and promoted through a Scottish hand therapy distribution list, a hand therapy WhatsApp group, a national brachial plexus therapy WhatsApp group and personal social media accounts of the authors (X, LinkedIn). Personal contacts/networks of the study team were also invited to participate and the team encouraged snowballing to enhance recruitment. Reminders were sent weekly via social media and with 1 week to go to email contacts. The study was open from 14/03/2025 to 01/05/2025 (7 weeks).

### Sample size

It was challenging to estimate population and sample size due to a lack of published data on rehabilitation of adults with a traumatic peripheral nerve injury. Peripheral nerve injuries are relatively uncommon with the average incidence of peripheral nerve injury in England 11.2 (95% CI 10.9–11.6) events per 100,000 population.^6^ We based our samples size calculation on UK specialist interest group membership (BAHT = 760, ATOCP = 600), the specialist nature of the condition and an assumption that not all therapists treat adults with traumatic nerve injury. From clinical experience we estimated that 20% of therapists treated adults with traumatic nerve injury. It was therefore estimated that there was a population of approximately 240 eligible therapists. Commonly reported response rated for online surveys in this cohort vary between 10–20%.^18,20^ We aimed for a 20% response rate (target 50) which we deemed sufficient to address our questions.

### Analysis

Data were analysed descriptively using Microsoft Excel (Microsoft 365). This included summarising the demographic characteristics of the respondents and the frequency of responses to dichotomised questions. The distribution of responses for Likert-type questions were analysed individually and then grouped into “always, frequently” to indicate those used frequently and “rarely, never” to indicate those used infrequently. Any free text responses to additional treatment techniques were extracted from the survey and grouped, counted and reported in additions to original items in the Likert scale (supplemental material).

We reported our findings in accordance with the CROSS reporting checklist^15^ (supplemental material 2). 

## Results

### Sample characteristics

A total of 53 participants completed the survey. The average time taken to complete the survey was 12 min and 30 s. Given the multiple routes to dissemination of the survey it was not possible to estimate response rate. Sixty percent (32/53) of respondents were physiotherapists, 40% (21/53) were occupational therapists, with 55% (29/53) working in Hand Therapy. A further 21% (11/53) of respondents were from trauma and orthopaedic outpatients and 15% (8/53) from NHS specialist nerve units. Most respondents were senior professionals, Agenda for Change Band 7, equivalent or above (*n* = 47/53).

Seventy percent of respondents (37/53) had been treating adults with nerve injuries for more than 10 years with only two respondents treating nerve injuries for 2 years or less. Ninety eight percent of respondents (52/53) treated adults with upper limb nerve injuries, with 68% (36/53) treating only upper limb injuries, with 30% (16/53) treating lower limb nerve injuries in addition to upper limb injuries and only one respondent treated adults with lower limb nerve injuries only. (Sample characteristics are detailed in Table 1).

**Table 1.** Respondent characteristics.

Respondent characteristics	Number of responses (%)
Profession	
Physiotherapist	32 (60)
Occupational therapist	21 (40)
Professional working area	
NHS hand therapy unit	28 (53)
Private practice (hand therapy)	1 (2)
NHS tertiary nerve injury unit	8 (15)
NHS trauma and orthopaedic outpatients	11 (21)
NHS trauma and orthopaedic inpatients	3 (6)
NHS burns and plastics	1 (2)
NHS community care	1 (2)
AfC^a^ banding	
6	6 (11)
7	27 (51)
8a/b Or equivalent in private practice	20 (38)
Clinical experience	
0–2 years	2 (4)
3–5 years	6 (11)
6–10 years	8 (15)
10 years plus	37 (70)
Types of nerve injury	
Isolated upper limb	36 (68)
Isolated lower limb	1 (2)
Combination upper and lower limbs	16 (30)

^a^AfC: Agenda for change.

Twenty three percent of respondents (12/53) saw more than 4 new patients with nerve injuries a month with 57% (30/53) seeing at least 1–2 new patients a month. Despite relatively low numbers of new patients presenting with peripheral nerve injury respondents reported seeing patients for long periods of rehabilitation with 62% of respondents (33/53) reporting that they saw patient up to 18 months after injury with 37% of respondents (20/53) reporting that they saw these patients for at least 2 years after their injury.

### Guidance

Twenty one percent of respondents (11/53) documented that their unit had written guidance/protocols for rehabilitation of all adults after nerve injury. Forty five percent of respondents (24/53) reported their unit only had guidance for post operative care with one respondent reporting that they had guidelines only for patients not needing surgery. Thirty-two percent of respondents (17/53) reported there were no guidance for adults after nerve injury.

### Frequency of treatment modalities used before and after motor or sensory reinnervation

Across both pre- and post-reinnervation phases, rehabilitation strategies such as passive and active range of movement, splinting, functional activity practice, and facilitation of self-management were reported as “always/frequently” used by over 80% of respondents. In contrast, several interventions were infrequently used, with consistently high “always/frequently” ratings. These included electrical stimulation (≤11%), thermal desensitisation (≤40%), and all psychological therapies (See Table 2).

**Table 2.** Reported current rehabilitation strategies for adult peripheral nerve injury.

	Pre- motor or sensory innervation *N* (%)			Post -motor or sensory innervation *N* (%)		
	Always/Frequently	Occasionally	Rarely/Never	Always/Frequently	Occasionally	Rarely/Never
Passive range of movement	53 (100)	0 (0)	0 (0)	49 (92)	4 (8)	0 (0)
Active range of movement	45 (85)	4 (8)	4 (8)	52 (98)	1 (2)	0 (0)
Strength exercises	28 (53)	14 (26)	11 (21)	50 (94)	2 (4)	1 (2)
Functional activity practice	43 (81)	7 (13)	3 (6)	52 (98)	1 (2)	0 (0)
Tactile desensitisation	41 (77)	7 (13)	5 (9)	43 (81)	9 (17)	1 (2)
Thermal desensitisation	14 (26)	15 (28)	24 (45)	21 (40)	15 (28)	17 (32)
Mental/Motor imagery	40 (75)	11 (21)	2 (4)	36 (68)	15 (28)	2 (4)
Mirror therapy	19 (36)	23 (43)	11 (21)	18 (34)	25 (47)	10 (19)
Localisation of touch	22 (42)	16 (30)	15 (28)	28 (53)	16 (30)	9 (17)
Texture and shape discrimination	21 (40)	13 (25)	19 (36)	31 (58)	15 (28)	7 (13)
Electrical stimulation	5 (9)	4 (8)	44 (83)	6 (11)	8 (15)	39 (74)
Vocational support	21 (40)	18 (34)	14 (26)	29 (55)	14 (26)	10 (19)
Facilitation of self-management	43 (81)	9 (17)	1 (2)	46 (87)	6 (11)	1 (2)
Splinting	47 (89)	4 (8)	2 (4)	47 (89)	4 (8)	2 (4)
Pain neuroscience education	38 (72)	9 (17)	6 (11)	38 (72)	8 (15)	7 (13)
Interdisciplinary pain management	26 (49)	19 (36)	8 (15)	24 (45)	20 (38)	9 (17)
Neural regeneration education	44 (83)	7 (13)	8 (15)	40 (75)	8 (15)	5 (19)
Cognitive behavioural techniques	14 (26)	16 (30)	23 (43)	15 (28)	13 (25)	25 (47)
Acceptance commitment therapy	11 (21)	8 (15)	34 (64)	12 (23)	12 (23)	29 (55)
Mindfulness	9 (17)	14 (26)	30 (57)	9 (17)	14 (26)	30 (57)

Comparing frequencies of pre- and post-reinnervation treatments for nerve injury revealed some differences. Motor-focused interventions such as active range of movement (from 85% to 98% always/frequently used), strength exercises (53% to 94%), and functional activity practice (81% to 98%) showed increased frequency of use post reinnervation. Respondents also reported an increased frequency in sensory rehabilitation post reinnervation, particularly texture and shape discrimination (40% to 58%) and localisation of touch (42% to 53%). Thermal desensitisation and electrical stimulation remained less frequently used both before and after reinnervation, though there were modest increases post-injury. Interventions related to pain management and long-term self-management such as vocational support, self-management, and pain neuroscience education, either increased slightly or remained stable in use.

**Figure 1.**
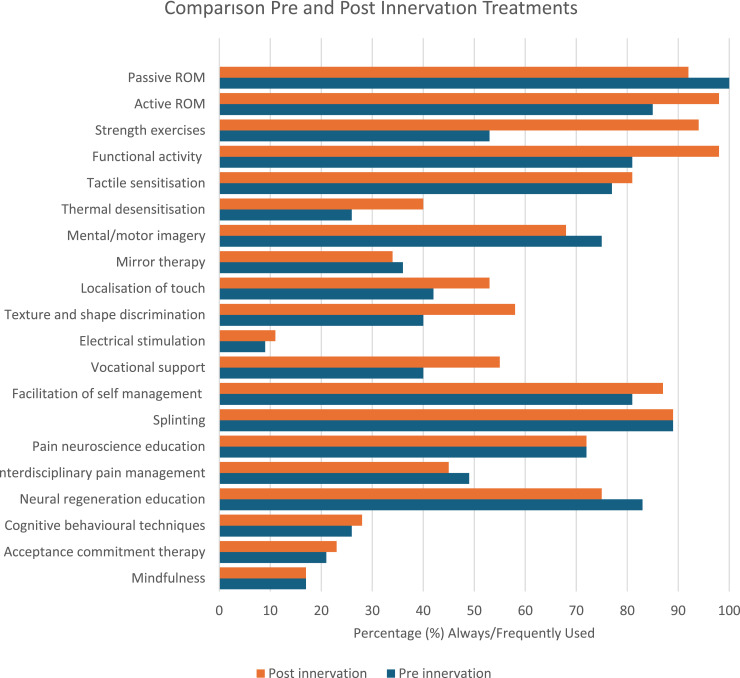
Comparison of pre and post reinnervation treatments. *ROM: Range of movement.

Analysis illustrated that psychologically informed therapies, including cognitive behavioural techniques (CBT), acceptance and commitment therapy (ACT), and mindfulness, were infrequently used by therapists both pre and post reinnervation, with “always/frequently” use under 30% across all three approaches (Figure 1).

### Organisational barriers to rehabilitation

**Figure 2.**
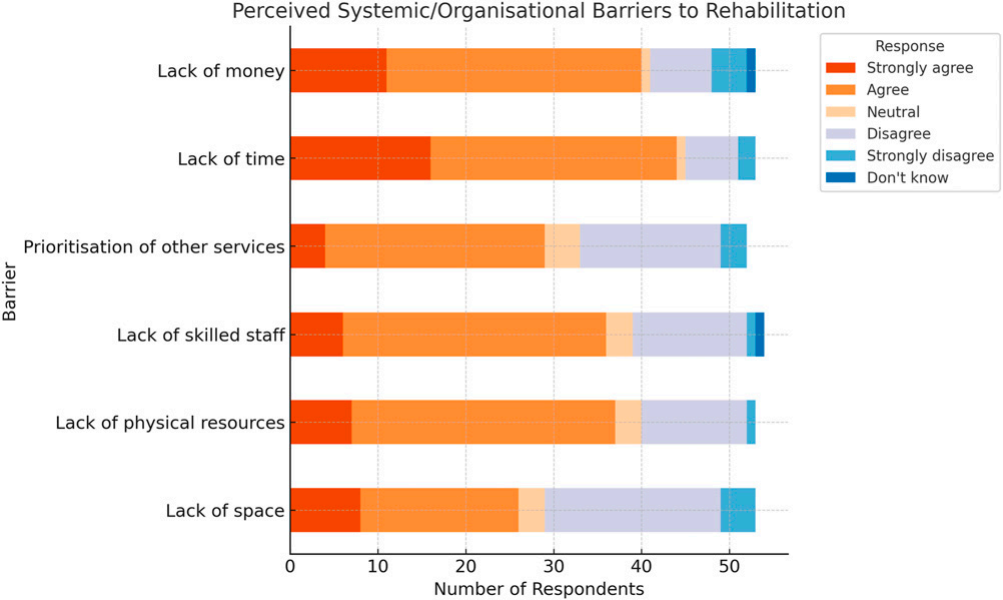
Perceived systemic/organisational barriers to rehabilitation.

Time, money, skilled staff, and physical resources were the most agreed-upon constraints to providing care. Prioritisation of other services and lack of space are more contested, with a higher number of respondents disagreeing or expressing neutrality (Figure 2).

### Psychological support

Fifty five percent of respondents (*n* = 29/53) reported adults attending for outpatient rehabilitation with nerve injury did not have access to Clinical Psychology professionals. For respondents who had access to Clinical Psychology support for adults with nerve injury (in an outpatient setting) thirty percent (*n* = 7/23) reported adults could wait on average a month with an additional 22% (*n* = 5/23) of respondents reporting adults could wait for 3 months. With regards access as an inpatient to a Clinical Psychology professional, 40% (*n* = 21/53) of respondents reported that there was no access and for those that had access 50% (*n* = 16/32) of respondents were unclear about the waiting times.

All respondents (100%) reported that some or all adults with nerve injury should have psychological support. Most respondents (83%, *n* = 44/53) felt that physiotherapists or occupational therapists could develop skills to deliver more psychologically informed rehabilitation with a further 15% (*n* = 8/53) responding “maybe”. Respondents believed adults with a nerve injury would most likely use a mobile app (96%, *n* = 51/53) followed by a web-based platform (72%, *n* = 38/53) or paper-based information (72%, *n* = 38/53) to support their rehabilitation.

### Involvement in future research

Sixty-eight percent of therapy respondents (*n* = 36/53) highlighted they would be interested in being involved further research investigating how to best support adults with nerve injury in the future.

## Discussion

This UK survey provides an insight into the current rehabilitation practices for adults with peripheral nerve injury in the UK. It was completed by very experienced therapists with 70% treating adults with nerve injury for 10 years or more. It highlights the consistency of core biomedically based motor and sensory treatment strategies, limited access to psychological support and key systemic challenges surrounding rehabilitation for adults with nerve injury.

Motor-focused rehabilitation remains central to practice, with near-universal use of active and passive range of motion, splinting, strength training, and functional activity. These reflect standard components of motor and functional recovery pathways**.** Comparing frequencies of pre- and post-reinnervation treatments for nerve injury revealed some differences. Motor-focused interventions such as active range of movement (from 85% to 98% always/frequently used), strength exercises (53% to 94%), and functional activity practice (81% to 98%) showed increased frequency of use post reinnervation. The increased frequency of these treatments post-reinnervation reflects the clinical emphasis on functional restoration once motor or sensory recovery begins. This is consistent with UK hand therapy national guidelines advocating initial protection followed by progressive mobilisation and muscle retraining^21^. 

However, the high proportion of respondents reporting the use of active range of motion (85%) and strength training (53%) prior to reinnervation is unexpected, as muscles affected by motor denervation would not have functional contractile capacity at this stage. This may suggest that clinicians are targeting adjacent or unaffected muscles rather than denervated ones directly. Current BAHT guidelines^21^ support active exercises and muscle retraining but do not distinguish between pre- and post-innervation phases, which may contribute to variation in practice. Importantly, the boundary between these phases may not be clear-cut. Motor reinnervation often occurs earlier than sensory reinnervation and requires fewer nerve fibres to restore some function, whereas sensory recovery is more complex and slower. It is possible that some therapy is delivered when partial motor reinnervation has already begun, even if sensory recovery lags. These biological differences, along with uncertainty about timing, may influence clinical decisions and explain why interventions appear to overlap across phases. Sensory-specific strategies such as texture discrimination and localisation of touch were used more frequently post-reinnervation, aligning with neuroplasticity principles and sensory re-education frameworks.^22^

In adults with traumatic life-changing nerve injuries, there is a growing recognition of the impact psychological factors play in outcome.^23^ The National Institute for Health and Care Excellence guideline^10^ for rehabilitation after traumatic injury with nerve injury (NG211) recommend physical therapy and psychological support. The recent James Lind Alliance Priorities Setting Framework for Brachial Plexus Injuries ranked psychological treatments as the number one priority for research.^24^ Despite all respondents in this study recognising the need for psychological support, over half reported no access to Clinical Psychology when patients are outpatients. Where access existed, long wait times and unclear referral pathways were common. These findings suggest a disconnect between clinical recognition of psychosocial need and systemic capacity to address it.

Psychologically informed practice, which integrates physical, behavioural, and psychological interventions, is increasingly advocated as a biopsychosocial intervention for patients with musculoskeletalpain.^25^ Almost one third of respondents (28%) occasionally/rarely supported adults with nerve injury with pain neuroscience education which is a key component in psychological support. This may link to a lack in pain science knowledge highlighted in a recent survey of therapists in this area.^26^ However, 84% of respondents supported patients to self-manage across all stages of rehabilitation, potentially mediating self-efficacy, a key predictor of psychological outcomes.^27^ More specific psychologically focussed approaches including CBT, ACT, and mindfulness were used rarely by the respondents in the study. These findings echo similar gaps identified in complex trauma rehabilitation^28^ where psychological distress can significantly affect outcomes.^29,30^ However, the majority of respondents in this study reported they felt they could develop skills to deliver more psychologically informed rehabilitation.

There are organisational and systemic barriers which further compound the above challenges. Lack of time, money, physical resources, and skilled staff were the most frequently reported obstacles to rehabilitation. These barriers are consistent with broader workforce pressures in rehabilitation services across the NHS.^31^ The lack of local clinical guidance, reported by a third of respondents, adds to variation in practice and may hinder the implementation of evidence-based interventions. The results, however, do highlight a strong appetite for innovation and research, with most respondents expressing interest in further involvement. Respondents also believed that patients with nerve injuries would use digital support platforms, particularly mobile apps and web-based tools, which may offer scalable solutions to address gaps in education and self-management support. This finding aligns with recent perspectives from adults with severe upper limb nerve injuries (in Sweden) where patients highlighted the need for digital solutions to support rehabilitation.^14^

This study has some limitations. Firstly, as a cross-sectional survey, findings reflect a snapshot in time and may not account for evolving rehabilitation practices. The self-selecting sample and recruitment via professional networks may have introduced selection bias, with respondents potentially more engaged or experienced than the wider workforce. There were 53 survey respondents in the cross-sectional survey. This low sample size is likely to under-represent the number of UK physiotherapists and occupational therapists who rehabilitate adults with nerve injury. A higher response rate might have been achieved by directly targeting individual NHS Hand Therapy and Trauma rehabilitation units across the UK. However, approaching NHS staff directly would likely have required full NHS ethical approval, which was beyond the resources, scope and timeframe of this survey. The use of a non-validated questionnaire, though developed through expert input and piloting, may affect the reliability and consistency of the data collected. Additionally, while the survey was designed to be anonymous and inclusive, the absence of identifiable data limited the ability to verify responses or prevent duplication.

Finally, we were unable to collect data on the geographical distribution of respondents. Although we initially included a question that allowed participants to indicate their region, this was not approved by the ethics panel, and the item was subsequently removed from the final survey. Without geographic information, we are unable to determine whether responses were distributed evenly across regions or whether certain areas were over- or under-represented. As a result, the sample may not reflect the wider national population, and findings should therefore be interpreted with caution. This lack of representativeness may limit generalisability and could obscure potential regional differences in practice and service provision.

This study suggests that rehabilitation for adults with peripheral nerve injury remains largely biomedical, despite therapists recognising the need for psychological support. Barriers such as limited time, skills access to Clinical Psychology and unclear referral pathways likely contribute to this gap. There is evidence that therapists may lack confidence and knowledge in modern pain science.^26^ Stern & Howe's 2021 paper reported limitations in understanding pain neurophysiology and reduced confidence in applying psychological strategies in hand therapists working in United States of America, particularly in complex cases.^26^ Importantly, increased knowledge was associated with greater confidence in evaluation and treatment, supporting the need for targeted training.^26^ Therefore, psychologically informed rehabilitation may require both better access to specialist psychological services and structured opportunities for skill development. A broader cultural change, shifting from biomedical to biopsychosocial will support this. In other non-acute areas of rehabilitation, there has been success in implementing this change. The Back Skills Training (BeST) programme for persistent low back pain showed that online training can shift clinicians’ attitudes and improve confidence, though organisational barriers impacted some uptake.^32^ Similar strategies combined with leadership support and practical delivery models could help embed biopsychosocial principles in acute nerve injury rehabilitation.

In conclusion, the study provides an important foundation for improving the quality of traumatic peripheral nerve injury rehabilitation in the UK. Biopsychosocial rehabilitation is limited, and this gap highlights a need for more integrated, biopsychosocial rehabilitation models, ensuring mental health and emotional adjustment are actively supported alongside physical recovery. The study included a broad study population of adults with traumatic peripheral nerve injury. Future research should focus on specific areas identified, including differences between upper and lower limb injuries, and exploration of how indicators such as injury severity and psychological impact predict rehabilitation needs and outcomes. Finally, evaluating the effectiveness of biopsychosocial training and exploring the role of digital tools in supporting long-term recovery for adults with nerve injury should be a key priority for future research.

## Supplemental material

**Supplemental material -** Current rehabilitation for adults with peripheral nerve injuries in the UK: An online survey Supplemental material Current rehabilitation for adults with peripheral nerve injuries in the UK: An online survey by Caroline Miller, Suzanne Beale, Beth Fordham, David J. Keene, Kenneth F. Valyear in Hand Therapy

**Supplemental material -** Current rehabilitation for adults with peripheral nerve injuries in the UK: An online survey Supplemental material Current rehabilitation for adults with peripheral nerve injuries in the UK: An online survey by Caroline Miller, Suzanne Beale, Beth Fordham, David J. Keene, Kenneth F. Valyear in Hand Therapy
