# Supplementary material for: Current rehabilitation for adults with peripheral nerve injuries in the UK: An online survey
Source: Hand Ther. 2026 Feb 4:17589983261420313. Online ahead of print. doi: 10.1177/17589983261420313 (PMC12872429; doi:10.1177/17589983261420313)
Supplement: Supplemental material - Current rehabilitation for adults with peripheral nerve injuries in the UK: An online survey [file sj-pdf-1-hth-10.1177_17589983261420313.pdf]

30 minutes

Current rehabilitation for adults with peripheral nerve injuries in the UK

Thank you for completing this survey. We are scoping current rehabilitation practice for adults with peripheral nerve injuries the UK.

Who can complete the survey?
Any registered healthcare professional who treats/ rehabilitates people with nerve injuries in the UK.

How long will the survey take?
The survey will take about 10-12 minutes
Participant information Sheet: To view the full participant information sheet please click this link: https://bham-my.sharepoint.com/

* Required

Eligibility

Please confirm your eligibility to take part in the survey

1. I am a UK based rehabilitation professional (physiotherapist or occupational therapist) who has experience of treating adults with peripheral nerve injury *

- ◯ Yes
- ◯ No

About You

2. Which of the following best describes your professional background?

- ◯ Occupational Therapist
- ◯ Physiotherapist
- ◯ Other

3. What is your area of clinical work?

- ◯ NHS - Hand Therapy Unit
- ◯ NHS - Trauma and Orthopaedics (outpatients)
- ◯ NHS - Trauma and Orthopaedics (inpatients)
- ◯ NHS - Specialist Nerve Clinic
- ◯ NHS - Pain management
- ◯ NHS community care
- ◯ NHS tertiary care (please specify)
- ◯ Private practice (please specify)
- ◯ Other

4. If you work in the NHS what is your current banding?

If you are not on an Agenda for Change banding then please add your title to the other section

- Band 4
- Band 5
- Band 6
- Band 7
- Band 8a
- Band 8b
- Band 8c or higher
- N/A
- Other

5. How many years have you been treating adults with a nerve injury?

- 0-2 years
- 3-5 years
- 6-10 years
- 10+ years

Experience of treating adults with a peripheral nerve injury

This section aims to gather information about your experience of treating adults with a peripheral nerve injury. A traumatic nerve injury is defined as any traumatic injury to the peripheral nerves excluding digital nerve injuries.

6. In which of the following body regions do you commonly see adults with a peripheral nerve injury?

- ◯ Isolated upper limb
- ◯ Isolated lower limb
- ◯ A combination of the above

7. As an estimate how many **new patients** with a peripheral nerve injury do you personally **assess per year?**

- ◯ Less than 12 (< 1 a month)
- ◯ 12-24 (1-2 a month)
- ◯ 25-36 (2-3 per month)
- ◯ 37-48 (3-4 per month)
- ◯ 50-99 (4-8 per month)
- ◯ 100+ (> 8 a month)

8. As an estimate how many **follow-up patients** with a peripheral nerve injury do you personally **see per year?**

- Less than 12 (< 1 a month)
- 12-24 (1-2 a month)
- 25-36 (2-3 a month)
- 37-48 (3-4 per month)
- 50-99 (4-8 per month)
- 100+ (>8 a month)

9. On average, how long do you have adults with peripheral nerve injury (excluding digital nerve injuries) as part of your caseload?

- 0-1 month
- 2-3 months
- 4-6 months
- 7-12 months
- 13-18 months
- up to 2 years
- up to 3 years
- up to 5 years
- Other

10. Does your centre have treatment guidance/protocol for treating adults with a traumatic peripheral nerve injury?

- ◯ Yes, for post operative patients only
- ◯ Yes, for patients not having surgery only
- ◯ Yes, for all patients
- ◯ No guidance/ protocol

11. Rate the following treatment options in terms of how commonly you use them or aspects of them when treating patients with an **acute peripheral nerve injury and BEFORE motor or sensory reinnervation.** *

	Always	Frequently	Occassionally	Rarely	Never
Passive range of motion exercises	◯	◯	◯	◯	◯
Active range of motion exercises	◯	◯	◯	◯	◯
Strength exercises	◯	◯	◯	◯	◯
Functional activity practice	◯	◯	◯	◯	◯
Tactile desensitisation	◯	◯	◯	◯	◯
Thermal desensitisation	◯	◯	◯	◯	◯
Mental/Motor imagery	◯	◯	◯	◯	◯
Mirror therapy	◯	◯	◯	◯	◯
Localisation of touch	◯	◯	◯	◯	◯
Texture and shape discrimination	◯	◯	◯	◯	◯
Electrical stimulation	◯	◯	◯	◯	◯
Vocational support	◯	◯	◯	◯	◯

	Always	Frequently	Occassionally	Rarely	Never
Facilitation of self management	○	○	○	○	○
Splinting	○	○	○	○	○
Pain neuroscience education	○	○	○	○	○
Interdisciplinary pain managment	○	○	○	○	○
Neural regeneration education	○	○	○	○	○
Cognitive behavioural techniques	○	○	○	○	○
Acceptance commitment therapy techniques	○	○	○	○	○
Mindfulness techniques	○	○	○	○	○

12. Are there any additional rehabilitation strategies you use with adults following an acute peripheral nerve injury **BEFORE motor or sensory reinnervation?**

13. From the list of treatment options in the previous question are there any which in your view are ineffective and/unsafe for use with adults with **acute peripheral nerve injuries BEFORE sensory or motor reinnervation?** Please describe your reasons for this.

14. Rate the following treatment options in terms of how commonly you use them or aspects of them when treating patients with an **acute peripheral nerve injury and AFTER motor or sensory reinnervation.** *

	Always	Frequently	Occassionally	Rarely	Never
Passive range of motion exercises	◯	◯	◯	◯	◯
Active range of motion exercises	◯	◯	◯	◯	◯
Strength exercises	◯	◯	◯	◯	◯
Functional activity practice	◯	◯	◯	◯	◯
Tactile desensitisation	◯	◯	◯	◯	◯
Thermal desensitisation	◯	◯	◯	◯	◯
Mental/Motor imagery	◯	◯	◯	◯	◯
Mirror therapy	◯	◯	◯	◯	◯
Localisation of touch	◯	◯	◯	◯	◯
Texture and shape discrimination	◯	◯	◯	◯	◯
Electrical stimulation	◯	◯	◯	◯	◯
Vocational support	◯	◯	◯	◯	◯

	Always	Frequently	Occassionally	Rarely	Never
Facilitation of self management	○	○	○	○	○
Splinting	○	○	○	○	○
Pain neuroscience education	○	○	○	○	○
Interdisciplinary pain managment	○	○	○	○	○
Neural regeneration education	○	○	○	○	○
Cognitive behavioural techniques	○	○	○	○	○
Acceptance commitment therapy techniques	○	○	○	○	○
Mindfulness techniques	○	○	○	○	○

15. From the list of treatment options in the previous question are there any which in your view are ineffective and/unsafe for use with adults with **acute peripheral nerve injuries AFTER sensory or motor reinnervation?** Please describe your reasons for this.

16. From the list of treatment options in the previous question are there any which in your view are ineffective and/unsafe for peripheral nerve injuries which have started to reinnervate? Please describe your reasons for this.

Current perceptions of systems barriers to rehabilitation

17. Do you perceive these items as barriers to rehabilitation for adults with peripheral nerve injury in your service ? *

	Strongly agree	Agree	Disagree	Strongly disagree	Neutral	Don't know
Lack of money/funding	◯	◯	◯	◯	◯	◯
Lack of time	◯	◯	◯	◯	◯	◯
Prioritsation of other services	◯	◯	◯	◯	◯	◯
Lack of qualified staff	◯	◯	◯	◯	◯	◯
Lack of physical resources or difficulty with existing resources	◯	◯	◯	◯	◯	◯
Lack of space	◯	◯	◯	◯	◯	◯

18. Do adults with a peripheral nerve injury (excluding those with a digital nerve injury)have access to psychological support (clinical psychologist or other mental health support) **as an inpatient** at your centre? *

- ◯ Yes
- ◯ No
- ◯ Unsure
- ◯ Other

19. How long do adults with a peripheral nerve injury need to wait to see a clinical psychologist or recieve other mental health support **as an inpatient** at your centre? *

- ◯ less than 24 hours
- ◯ 2-3 days
- ◯ 4 days or more
- ◯ 5 days or more
- ◯ Unsure

20. Do adults with a peripheral nerve injury (excluding digital nerves) have access to psychological support (clinical psychologist or other mental health support) **as an outpatient** at your centre? *

- ◯ Yes
- ◯ No
- ◯ Unsure
- ◯ Other

21. How long do adults with a peripheral nerve injury wait to see a clinical psychologist or recieve other mental health support **as an outpatient** at your centre?

- less than 24 hours
- 2-3 days
- 4-5 days
- less than a week
- 8-28 days
- 1-3 months
- 4-6 months
- 7 months or longer
- Unsure

22. Which patients with a peripheral nerve injury should have access to psychology support ? *

- All patients
- Some patients
- Access is not important
- Other

23. Do you think that physiotherapists, occupational therapists and nurses can develop skills to deliver more advanced psychological support (such as acceptance and commitment therapy) as part of their rehabilitation of patients with acute nerve injuries? *

- ◯ Yes
- ◯ No
- ◯ Maybe
- ◯ Other

24. We would like to understand more about your response to the previous question. Please enter any reasoning behind your answer below.

25. Do you think that the adults you see with a nerve injury would use the following to support their overall care after a nerve injury? *

Click all options which you think are relevant

- ☐ Mobile application (APP)
- ☐ Web based platform
- ☐ Paper based information
- ☐ Wearable device (to monitor limb movement)
- ☐ Other

26. Would you be interested in being involved in future research investigating how we best support adults with a peripheral nerve injury in the future? *

- ◯ Yes
- ◯ No
- ◯ Maybe
- ◯ Other

Contact the team

Please contact Caroline Miller at Caroline.miller@uhb.nhs.uk if you would like to be involved in future research with adults with nerve injuries

End of Form

Thank you very much for taking part in this survey. We appreciate the time given to complete it.

We are sorry you are not eligible to complete this form.

Thank you for attempting to complete our survey but unfortunately you are not eligible to complete this piece of research.

This content is neither created nor endorsed by Microsoft. The data you submit will be sent to the form owner.

Microsoft Forms
